# Physiological and Biochemical Characters of Eight Native Tree Seedings in Guangdong Province During Drought Stress and Rewatering Treatment

**DOI:** 10.3390/plants15040528

**Published:** 2026-02-08

**Authors:** Qiutong Liu, Zhihong Liu, Jingquan Liu, Kexin Li, Jieyu Lin, Shufan Lin, Zijia Su, Xinyi Fan, Yv Su, Zujing Chen

**Affiliations:** 1 College of Forestry and Landscape Architecture, South China Agricultural University, Guangzhou 510642, China; 202218130120@stu.scau.edu.cn (Q.L.); 2967388693@stu.scau.edu.cn (Z.L.); jingquanliu@stu.scau.edu.cn (J.L.); 202218130113@stu.scau.edu.cn (K.L.); lbzfan@stu.scau.edu.cn (S.L.); fresity@stu.scau.edu.cn (Z.S.); 3317429282@stu.scau.edu.cn (X.F.); 2 College of Food Science, South China Agricultural University, Guangzhou 510642, China; 50003551@scau.edu.cn; 3 Guangzhou Institute of Forestry and Landscape Architecture, Guangzhou 510405, China; suyu110@163.com; 4 Guangzhou Collaborative Innovation Center on Science-Tech of Ecology and Landscape, Guangzhou 510405, China; 5 State Key Laboratory of Conservation and Utilization of Subtropical Agro-Bioresources, South China Agricultural University, Guangzhou 510642, China

**Keywords:** drought stress, water parameters osmotic solutes, antioxidant enzyme, photosynthesis, drought resistance evaluation

## Abstract

Native tree species play a crucial role in addressing the challenge of seasonal drought in South China. In this study, one-year-old seedlings of eight native tree species in Guangdong Province were subjected to continuous simulated drought stress and rewatering. In order to identify key drought-resistant traits and best performing tree species, physiological and biochemical responses were assessed through 21 indicators. The results showed the following: (1) All species exhibited responses to drought stress prior to the fourth day, as evidenced by reductions in morphological indicators (crown breadth and ground diameter) and photosynthetic parameters (chlorophyll content, transpiration rate, net photosynthetic rate and stomatal conductance), along with increases in osmotic substances (soluble protein and proline) and antioxidant-related indicators malondialdehyde, peroxidase and superoxide dismutase). (2) The crown breadth, leaf relative water content, chlorophyll content, and ascorbate peroxidase activity were significantly decreased under drought stress. And these indicators were not recovered to pre-stress levels following rewatering. (3) Mantel tests revealed that growth morphological characteristics, particularly plant height, were significantly and positively correlated with most osmotic substances indicators (*p* < 0.001). Specifically, plant height showed the strongest coupling with these traits, with Mantel’s *r* ranging from 0.44 to 0.89. In addition, the leaf relative water content, net photosynthetic rate, superoxide dismutase, and malondialdehyde were regarded as the key drought-resistant traits, providing insights into future research on plant improvement, stress-resilience breeding and even drought resistance mechanisms. (4) The eight tree species are ranked from most to least drought-resistant as follows: *Zenia insignis*, *Michelia macclurei*, *Phoebe zhennan*, *Phoebe bournei*, *Erythrophleum fordii*, *Dalbergia odorifera*, *Cinnamomum burmanni* and *Michelia chapensis*. This study provides a scientific basis for selecting tree species for afforestation in seasonally arid regions.

## 1. Introduction

Drought continues to be one of the most persistent and significant environmental stressors that restrict plant growth [1]. Anthropogenic climate change is intensifying the frequency and severity of extreme weather events in ecologically vulnerable regions [2,3]. Transitional climate zones, such as Guangdong Province in China, face intensified aridity due to long-term precipitation changes and higher evapotranspiration rates [4]. The southern coastal regions are particularly vulnerable to spring droughts because of insufficient rainfall, while the northern inland areas are more susceptible to autumn and winter droughts caused by the premature retreat of the summer monsoon [5]. Planting native tree species is one of the main solutions to address drought challenges in Guangdong Province [6].

Native tree species are characterized by low maintenance requirements, high environmental adaptability, and significant ecological functionality, such as *Schima superba* and *Michelia macclurei* [7,8,9]. The use of native trees confers ecological and economic benefits, including reduced procurement and maintenance costs and improved ecosystem stability through co-evolved traits and natural regeneration [10,11,12,13]. However, emerging empirical evidence increasingly challenges the resilience of native species to intensifying environmental perturbations. Recent studies highlight that certain native trees, such as *Fagus sylvatica* and *Picea abies*, have exhibited unexpected sensitivity and severe growth decline within their historical ranges due to intensifying drought cycles [14,15]. Therefore, understanding how trees perceive and mitigate drought stress is fundamental to developing a framework for quantitative assessment.

Drought stress profoundly affects plants at physiological, biochemical, and molecular levels [16]. Through long-term evolution, plants have developed three core drought resistance strategies: escape (accelerating the life cycle to avoid drought periods), avoidance (maintaining high tissue water potential to prevent damage), and tolerance (maintaining physiological function under low water potential) [17]. A representative example of drought escape is the desert shrub *Artemisia ordosica*, which employs water-saving tactics by shifting peak transpiration to earlier hours and tightly regulating stomatal conductance under limited soil moisture [18]. The distinction between drought avoidance and tolerance is especially evident among conifer seedlings: *Abies balsamea* adopts a conservative water-use strategy to avoid low water potential, while *Thuja occidentalis* can tolerate water potentials well below the critical hydraulic threshold, demonstrating a dehydration tolerance strategy [19]. Moreover, plants may integrate multiple strategies. For instance, *Quercus* spp. across precipitation gradients adapt to drought by simultaneously enhancing dehydration tolerance and dehydration avoidance capacity [20]. Therefore, classifying and quantitatively assessing various physiological and biochemical indicators of drought resistance constitutes a critical approach to evaluating tree species’ drought tolerance.

To comprehensively evaluate drought tolerance, the physiological and biochemical indicators of drought resistance can be categorized into four groups: morphological traits, osmotic regulation, photosynthetic characteristics, and metabolism. Plant height, ground diameter, and crown breadth serve as indicators of growth limitations [21]. Chlorophyll concentration (Chl), net photosynthetic rate (Pn) and transpiration rate (Tr) collectively reveal photosynthetic and hydraulic regulation strategies [22,23]. At the biochemical level, soluble protein (SP) content and soluble sugar (SS) acts as an osmotic regulator and energy source [24,25]. Proline (Pro) accumulation enhances drought tolerance; elevated malondialdehyde (MDA) indicates oxidative damage severity [3,26]. And increased superoxide dismutase (SOD), peroxidase (POD), ascorbic acid peroxidase (APX) [27,28], and glutathione (GSH) levels signify activated antioxidant defenses [29,30]. Numerous studies have demonstrated that different protective enzymes exhibit distinct responses to drought stress, and the enzymes playing a dominant role in plants may vary across different stages of stress [31].

Research on the drought resistance of native tree species primarily employs two main approaches: molecular studies and physiological and biochemical analysis [32,33]. At the molecular level, tree species respond to water deficit by activating intricate signaling cascades, primarily coordinated by abscisic acid (ABA)-dependent and -independent pathways [34]. Molecular studies focus on drought-responsive gene networks through transcriptome sequencing and functional validation [35,36]. These transcriptional shifts, involving key gene families such as *DREB*, *MYB*, and *LEA*, provide the regulatory blueprint for downstream physiological modifications [33,34]. Physiological and biochemical analysis focuses on examining the interrelationships among water loss, osmotic regulation, and antioxidant synergy. *Zenia insignis* resist drought stress by increasing root biomass to expand the area and distance of water absorption in soils lacking water [37]. *M. macclurei* reduces water loss and xylem embolism by regulating stomatal closure and decreasing turgor loss point [38]. *Phoebe bournei* activates the antioxidant defense system and mitigates oxidative stress through increased malondialdehyde (MDA) content in leaves and enhanced antioxidant enzyme activity [39]. *Phoebe zhennan* reduces water loss by decreasing leaf area, stomatal conductance, and transpiration rate, while enhancing water absorption through increased root biomass [40]. Despite progress in isolated mechanisms, we still lack quantitative frameworks capable of bridging the gap between transcriptomic data and multidimensional physiological responses [41,42]. Such an integrative synthesis is essential for developing a predictive model of forest resilience under global climate instability.

Based on the ecological constraints of seasonal drought in South China, this study aims to evaluate the drought resilience of eight native tree species through a multidimensional lens, encompassing 21 physiological and biochemical traits classified into morphological, permeability, photosynthetic, and antioxidant-related indicators. We hypothesized that (1) these native species differ significantly in their drought tolerance and recovery capacity, enabling quantitative data into a drought resistance hierarchy; (2) among the 21 indicators, certain key traits possess higher diagnostic value for assessing drought resistance than others. This work provides a multidimensional dataset for native tree species that integrates both drought resistance and recovery phases, thereby refining species selection criteria beyond simple survival rates. Furthermore, these findings provide a foundation for implementing precision afforestation strategies, enabling the selection of best tree species with synchronized resistance and recovery traits to enhance forest stability under a changing climate.

## 2. Results

### 2.1. Morphological Indicators

The reduction in height among eight species under drought stress varies between 1.0 and 2.5 cm (Table 1). The plant height of *Z. insignis* and *M. macclurei* showed an initial increase on D4 by 20.1% and 0.6%, respectively. *Cinnamomum burmanni* and *Z. insignis* exhibited limited growth recovery following rewatering. *Z. insignis*, *Michelia chapensis*, and *E.fordii* exhibited an initial increase in ground diameter on D4, followed by subsequent reduction. And final diameters remained below the initial values. Except for *Z. insignis*, which exhibited a 2.7% increase in ground diameter, the figures of all tree species decreased following drought stress and rewatering treatment. All eight species exhibited progressive crown area reduction throughout the drought period, and rewatering treatment failed to restore the damage. *P. zhennan* displayed the least reduction (63.8%), with other species experiencing 81.3-96.1% declines.

**Table 1:** Changes in plant height, ground diameter and crown breadth of eight native tree species under different drought stress and rewatering treatments. Values are presented as mean ± SEM.

Species	Processing Days	Morphological Indicators		
		Plant Height/cm	Ground Diameter/mm	Crown Breadth/cm^2^
*Zenia insignis*	D0	76.07 ± 1.90	5.09 ± 0.13	744.67 ± 45.00
	D4	77.60 ± 0.95	5.18 ± 0.10	409.28 ± 41.40
	D7	77.43 ± 1.03	5.00 ± 0.23	226.39 ± 33.33
	D14	76.67 ± 1.50	5.00 ± 0.44	128.86 ± 19.59
	R7	76.44 ± 1.31	5.07 ± 0.31	86.30 ± 4.60
	R14	76.41 ± 1.03	5.11 ± 0.32	86.30 ± 4.60
*Michelia macclurei*	D0	63.03 ± 0.83	4.77 ± 0.55	332.4 ± 33.99
	D4	63.47 ± 0.58	4.34 ± 0.54	65.18 ± 6.32
	D7	62.80 ± 0.52	4.12 ± 0.37	50.51 ± 3.85
	D14	62.53 ± 0.68	3.96 ± 0.45	42.33 ± 1.84
	R7	62.43 ± 0.59	3.92 ± 0.43	31.86 ± 11.26
	R14	62.07 ± 0.81	3.87 ± 0.39	42.72 ± 12.15
*Phoebe zhennan*	D0	48.57 ± 2.25	5.90 ± 0.82	197.81 ± 18.00
	D4	48.33 ± 2.45	5.80 ± 0.52	127.83 ± 13.42
	D7	47.93 ± 2.32	5.28 ± 0.53	80.48 ± 15.44
	D14	47.67 ± 2.32	4.72 ± 0.43	72.92 ± 28.29
	R7	46.53 ± 1.76	4.69 ± 0.34	74.15 ± 7.06
	R14	46.07 ± 1.46	4.61 ± 0.21	71.56 ± 10.66
*Phoebe bournei*	D0	46.27 ± 1.10	5.47 ± 0.57	262.96 ± 25.93
	D4	45.5 ± 0.92	5.07 ± 0.50	243.33 ± 23.68
	D7	45.47 ± 0.91	4.73 ± 0.12	82.71 ± 8.53
	D14	44.87 ± 1.29	4.70 ± 0.16	73.43 ± 5.52
	R7	44.47 ± 1.34	4.61 ± 0.54	31.22 ± 3.85
	R14	44.03 ± 0.55	4.71 ± 0.37	33.11 ± 2.27
*Erythrophleum fordii*	D0	60.28 ± 1.48	7.76 ± 0.58	1893.56 ± 153.4
	D4	60.23 ± 1.35	8.62 ± 1.22	1006.11 ± 78.34
	D7	59.80 ± 1.47	8.32 ± 1.65	860.83 ± 31.51
	D14	59.47 ± 1.52	8.03 ± 1.52	483.56 ± 13.14
	R7	59.10 ± 2.85	7.69 ± 1.25	353.95 ± 28.99
	R14	59.10 ± 2.85	7.77 ± 1.09	398.69 ± 43.79
*Dalbergia odorifera*	D0	61.93 ± 3.07	7.67 ± 0.47	526.87 ± 36.54
	D4	60.03 ± 2.15	6.73 ± 0.64	354.14 ± 26.90
	D7	59.17 ± 0.81	6.51 ± 0.12	219.62 ± 5.31
	D14	57.97 ± 1.75	6.16 ± 0.11	206.37 ± 12.04
	R7	57.10 ± 1.55	6.33 ± 0.33	60.61 ± 8.48
	R14	56.07 ± 0.98	6.37 ± 0.25	53.97 ± 8.10
*Cinnamomum burmanni*	D0	44.77 ± 1.66	6.18 ± 0.20	454.09 ± 58.74
	D4	44.17 ± 1.26	6.00 ± 0.26	267.00 ± 27.00
	D7	43.03 ± 0.70	5.68 ± 0.14	92.36 ± 42.53
	D14	42.53 ± 0.31	5.39 ± 0.52	71.60 ± 13.59
	R7	42.3 ± 0.26	5.22 ± 0.37	54.51 ± 7.26
	R14	42.33 ± 0.25	5.17 ± 0.43	74.51 ± 13.84
*Michelia chapensis*	D0	41.93 ± 0.42	5.87 ± 0.51	453.96 ± 22.32
	D4	40.43 ± 1.27	5.89 ± 0.80	178.37 ± 25.88
	D7	40.10 ± 0.79	5.79 ± 0.97	65.53 ± 15.94
	D14	40.06 ± 0.90	5.16 ± 0.84	47.89 ± 3.68
	R7	39.90 ± 0.61	4.87 ± 0.89	29.88 ± 5.09
	R14	39.43 ± 0.47	4.80 ± 0.92	17.84 ± 5.49

D0 = the day before the drought stress; D4, D7, D14 = the 4th, 7th, and 14th day of drought stress; R7, R14 = the 7th and 14th day of rewatering.

### 2.2. Soil Relative Water Content and Leaf Relative Water Content

**Figure 1:**
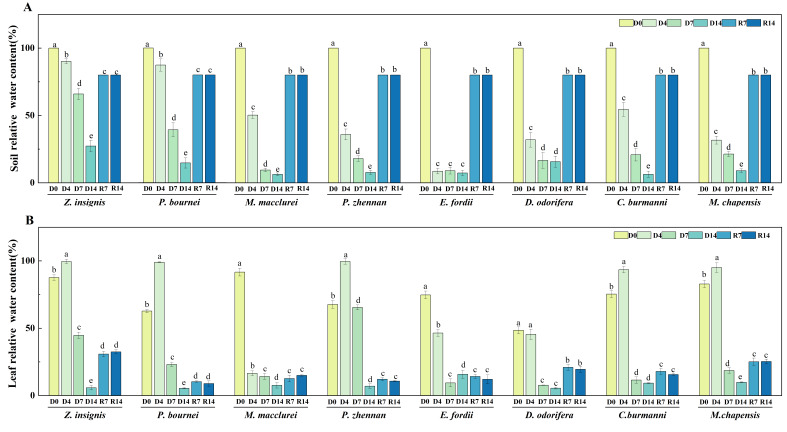
Effects of drought stress and rewatering on soil relative water content and leaf relative water content of eight native tree species in Guangdong Province. (**A**) Soil relative water content. (**B**) Leaf relative water content. D0 = the day before the drought; D4, D7, D14 = the 4th, 7th, and 14th day of drought stress; R7, R14 = the 7th and 14th day of rewatering. The date is presented as mean ± SEM (*n* = 15 seedlings per species per treatment, with three replicate measurements). Significant differences among treatments within each species were determined by Duncan’s multiple range test and are denoted by different lowercase letters.

In general, the rapid decrease in soil relative water content (SRWC) mainly occurs in the early stage of drought treatment (D4), and then the rate of SMC decline tends to be flat with the continuation of drought stress (Figure 1A). Baseline SRWC at D0 averaged 33.2–36.8% across species. *Z. insignis* has shown strong drought resistance. Its SRWC was 90.1% on D4 and remained at a high level of 27.3% on D14. Progressive soil desiccation occurred during drought stress, with species-specific minimum values at D14 ranging from 8.6% (*Erythrophleum fordii*) to 27.3% (*Z. insignis*). In contrast, the SRWC of *E. fordii* decreased significantly to 8.6% on D4. Except for *M. macclurei*, which exhibited a 75.1% decrease in leaf relative water content (LRWC) on D4, all tree species showed a significant decline in LRWC during the middle stage of drought (Figure 1B). *Z. insignis*, *P. zhennan*, *P. bournei*, *C. burmanni* and *M. chapensis* showed a brief increase on D4. The LPWC of *Z. insignis*, *Dalbergia odorifera* and *M. chapensis* remained at relatively high levels following rewatering treatment, reaching 32.4%, 21.0% and 25.3%, respectively.

### 2.3. Chlorophyll and Photosynthetic Parameters

**Figure 2:**
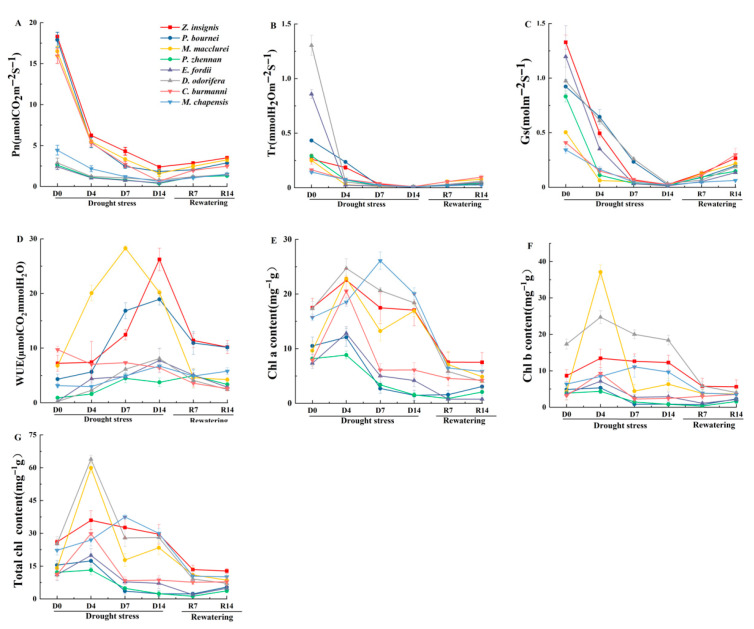
Effects of drought stress and rewatering on chlorophyll and photosynthetic parameters of eight native tree species in Guangdong Province. (**A**) Net photosynthetic rate. (**B**) Transpiration rate. (**C**) Stomatal conductance. (**D**) Water use efficiency. (**E**) Chlorophyll a. (**F**) Chlorophyll b. (**G**) Total chlorophyll. D0 = the day before the drought; D4, D7, D14 = the 4th, 7th, and 14th day of drought stress; R7, R14 = the 7th and 14th day of rewatering. Lines represent the means ± SEM, with *n* = 15 seedlings per species per treatment and three replicate measurements.

Under drought stress, the net photosynthetic rate (Pn), transpiration rate (Tr) and stomatal conductance (Gs) of all tree species showed a downward trend (Figure 2A–C). The Pn of *M. chapensis* and *C. burmanni* exhibited the most pronounced declines by D14, with decreases of 90.4% and 95.9%, respectively (Figure 2A). The Pn of *Z. insignis* recovered to 3.5 μmol CO_2_/m^2^/s at R14, and *E. fordii* increased Pn by 47.1%. *D. odorifera* showed the steepest declines of Tr, dropping from 1.3 to 0.1 mol/m^2^/s, a 99.2% decrease (Figure 2B). The Tr of *M. chapensis* and *Z. insignis* increased by 11.6% and 9.6% following rewatering treatment. The Gs of *Z. insignis* decreased most significantly under drought stress, with values ranging from 1.3 to 0.1 mol/m^2^/s (Figure 2C). Following rewatering treatment, the Gs of *Z. insignis* and *C. burmanni* exhibited noticeable recovery, whereas *M. chapensis* showed only a minimal recovery. The water use efficiency (WUE) values of all tree species increased under drought stress, while *Z. insignis*, *P. bournei*, and *M. chapensis* exhibited a transient decline on D4 (Figure 2D). *M. macclurei* reached a WUE peak at D7 (28.3 μmol/mmol) and *Z. insignis* peaked at D14, with increases of 263.5%. The chlorophyll a content of all eight tree species displayed a dynamic trend of initial increase followed by decrease under drought stress, with stable recovery patterns during rewatering (Figure 2E). *Z. insignis* and *D. odorifera*, increased by 31.4% and 42.7% on D4, respectively. The chlorophyll b content exhibited an initial increase followed by a subsequent decrease, with extreme fluctuations in *M. macclurei* and *D. odorifera* (Figure 2F). *D. odorifera* surged to 39.2 mg/g at D4 and dropped to 7.7 mg/g at D7. Total chlorophyll generally peaked on D4 or D7, followed by sharp declines under prolonged drought (Figure 2G). The total chlorophyll of *P. bournei* and *P. zhennan* decreased by 85.8% and 80.5% under drought stress, respectively.

### 2.4. Osmotic Solutes

**Figure 3:**
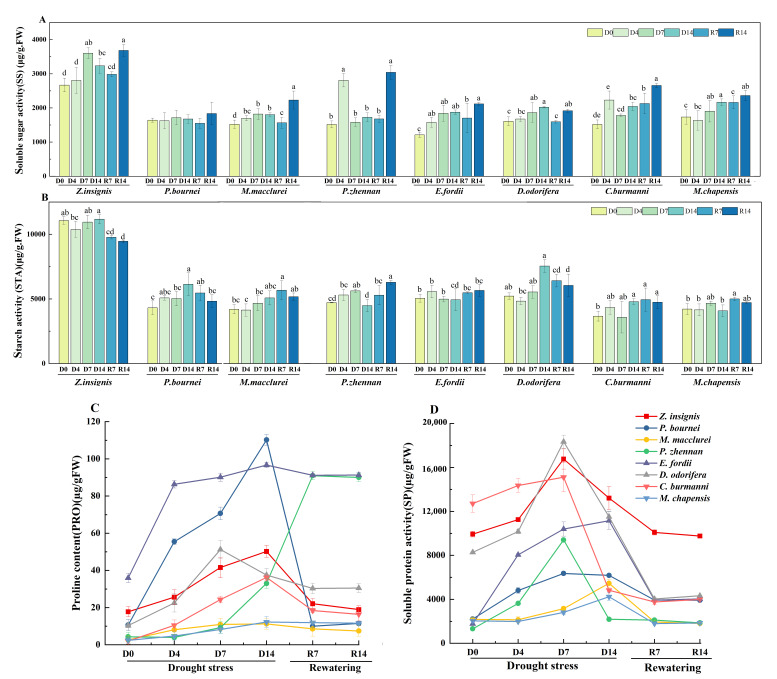
Effects of drought stress and rewatering on osmotic solute content of eight native tree species in Guangdong Province. (**A**) Soluble sugar. (**B**) Starch. (**C**) Proline. (**D**) Soluble protein. D0 = the day before the drought; D4, D7, D14 = the 4th, 7th, and 14th day of drought stress; R7, R14 = the 7th and 14th day of rewatering. The date is presented as mean ± SEM (*n* = 15 seedlings per species per treatment, with three replicate measurements). Significant differences among treatments within each species were determined by Duncan’s multiple range test and are denoted by different lowercase letters.

Under drought stress and rewatering treatments, significant differences were observed in starch (Sta) and soluble sugar (SS) accumulation among tree species. For Sta, *M. chapensis* exhibited an increase to 4663.0 μg/g on D7, followed by a decrease of 5.6% (Figure 3A). *C. burmanni* and *E. fordii* displayed relatively stable patterns. For SS, *C. burmanni* exhibited the most pronounced response, reaching a peak value of 2677.0 μg/g at R14 (Figure 3B). *P. zhennan* displayed fluctuations: its sugar content rose to 2830 μg/g at D4, sharply decreased by 27.2% to 1602.0 μg/g at D7, then increased by 30.3% to 3063.0 μg/g on R14. *P. bournei* showed the most significant increase in proline (Pro) content during drought, from 10.6 to 110.2 µg/gFW (Figure 3C). Following the rewatering treatment, the Pro content in *Z. insignis* and *P. zhennan* exhibited the most significant reductions, decreasing by 20% and 10%, respectively. Except for *M. macclurei*, the soluble protein (SP) of all species had reached a peak at D7, and the change range of *E. fordii* and *D. odorifera* was the largest (Figure 3D). In *M. macclurei* it reached its peak at D14, at 5455.6 µg/gFW.

### 2.5. Antioxidant Defense and Oxidative Damage

**Figure 4:**
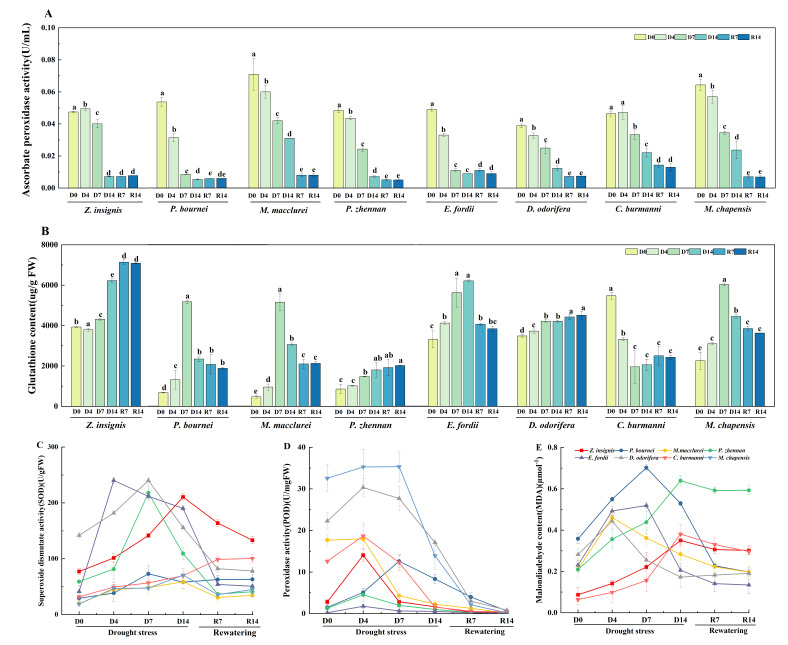
Effects of drought stress and rewatering on antioxidant enzyme activities, ascorbate peroxidase, glutathione, and malondialdehyde levels in eight native tree species. (**A**) Superoxide dismutase activity. (**B**) Peroxidase activity. (**C**) Malondialdehyde content. (**D**) Ascorbate peroxidase activity. (**E**) Glutathione content. D0 = the day before the drought; D4, D7, D14 = the 4th, 7th, and 14th day of drought stress; R7, R14 = the 7th and 14th day of rewatering. Dates are presented as mean ± SEM (*n* = 15 seedlings per species per treatment, with three replicate measurements). Significant differences among treatments within each species were determined by Duncan’s multiple range test and are denoted by different lowercase letters.

Under drought stress, the superoxide dismutase (SOD) activity in *P. zhennan*, *P. bournei*, *C. burmanni*, *E. fordii*, and *D. odorifera* initially increased and then decreased, while that in *M. macclurei*, *Z. insignis* and *M. chapensis* continued to rise (Figure 4A). *D. odorifera* reached its peak SOD on D7 at 240.1 U/gFW, with an increase of 69.2%. The SOD in *Z. insignis* decreased significantly following rewatering treatment, declining from 210.6 to 133.2 U/gFW. The peroxidase (POD) activity of all tree species, except for *P. bournei* and *M. chapensis*, reached its peak on the fourth day of drought stress (Figure 4B). *C. burmanni* maintained high POD throughout the drought period, with a 67.7% increase on D7. The malondialdehyde (MDA) content in *M. chapensis*, *M. macclurei*, *D. odorifera*, *P. bournei*, and *Z. insigni* increased initially and then decreased (Figure 4C). The MDA of *Z. insignis* and *P. bournei* peaked at D7, reaching 0.2 and 0.7 μmol/g. *P. zhennan* exhibited an increasing trend during drought stress, with the maximum value reaching 0.6 μmol/g. Under drought stress, the ascorbate peroxidase (APX) activity of eight tree species declined (Figure 4D). However, the APX in *Z. insigni* and *C. burmanni* increased by 4.0% and 1.7% on D4, respectively. Following rewatering treatment, the APX of *E. fordii* increased 24.0%. With the exception of *C. burmanni*, the glutathione (GSH) content in all tree species showed a continuous increase under drought stress (Figure 4E). *P. bournei* and *M. macclurei* exhibited the most pronounced increases, while *D. odorifera* demonstrated the highest relative content. During the drought stress, *Z. insigni* and *E. fordii* reached their peak levels on D14, with values of 6225.5 and 6180.4 µg/gFW, respectively.

### 2.6. Multivariate Statistical Analysis

**Figure 5:**
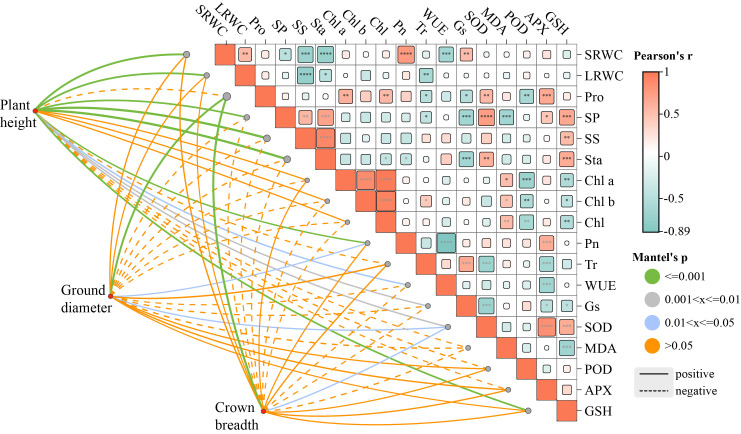
Correlation and Mantel analyses among 21 physiological and biochemical indexes of eight native tree species. Significance levels are denoted by * *p* < 0.05, ** *p* < 0.01, *** *p* < 0.001, and **** *p* < 0.0001. Color gradient represents correlation direction: red indicates positive correlation, while blue indicates negative correlation; green indicates a highly significant correlation, while orange denotes a non-significant relationship.

Growth-related morphological traits and photosynthetic indicators showed a strong correlation (Figure 5). Crown width decreased synchronously with ground diameter. LRWC was significantly positively correlated with SMC, and Pn showed a significant positive correlation with LRWC. Chlorophyll a, chlorophyll b, and total chlorophyll were strongly coupled with Pn. Within the antioxidant system, SOD and POD activities were synergistically upregulated (*p* ≤ 0.001), but both exhibited significant negative correlations with MDA levels. This indicates that a stronger reactive oxygen species scavenging capacity is associated with less severe membrane lipid peroxidation damage.

**Figure 6:**
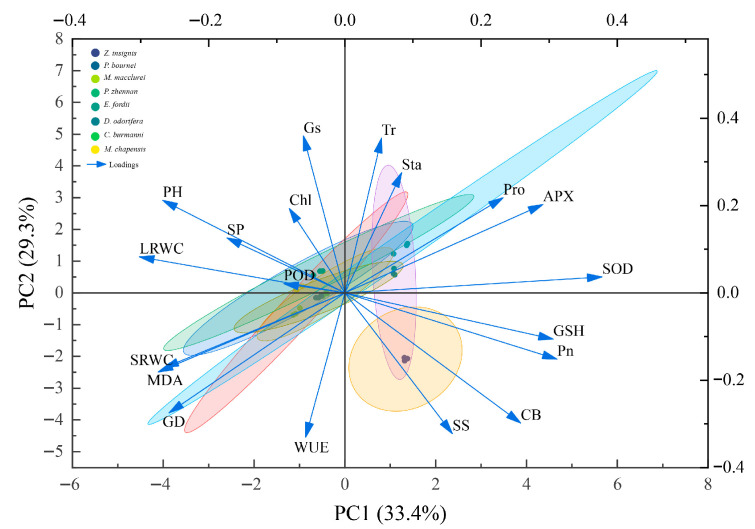
Principal component analysis biplot of eight tree species under drought stress and rewatering conditions. Each colored dot represents an individual sample replicate, and the colored ellipses denote the 95% confidence intervals for each of the eight tree species. Blue arrows represent the loading vectors of the physiological and growth indices. The direction and length of each arrow indicate the correlation and contribution of that trait to the principal components.

In total, 21 identification indicators under drought stress were classified into four principal components through principal component analysis. The contribution rates of the first four principal components were 33.4%, 29.3%, 13.8% and 8.5% respectively, with a cumulative contribution rate of 85.1%, thus retaining most of the original information (Table 2). The analysis of variable loadings revealed the trait specificity of each component (Table 3): Principal component 1 (CI_1_) had relatively high loadings on SOD, APX, GSH, leaf relative water content, Pn, and MDA, which mainly related to the leaf traits and antioxidant-related parameters. Principal component 2 (CI_2_) exhibited substantial loadings on Tr, Gs and WUE, being closely associated with photosynthesis. Principal components 3 (CI_3_) had significant loadings on POD. To visually verify these results, a PCA biplot (Figure 6) was constructed to display the relationships between indicators and components. Leaf relative water content, Pn, and MDA clustered near the CI_1_ axis, while Tr, Gs and WUE gathered around the CI_2_ axis.

The weight values of the four comprehensive indicators were 0.43, 0.29, 0.19 and 0.09, respectively (Table 4). Higher comprehensive evaluation values were positively correlated with stronger drought resistance ability. The results showed that *Z. insignis* had the largest value of 1.836, indicating the strongest drought resistance. The ranking from highest to lowest was as follows: *Z. insignis*, *M. macclurei*, *P. zhennan*, *P. bournei*, *E. fordii*, *D. odorifera*, *C. burmanni* and *M. chapensis*.

## 3. Discussion

The adaptive capacity of plants to survive drought, known as drought resistance, involves multi-level functional shifts and is classified into escape, avoidance, and tolerance mechanisms [17,42]. The eight native tree species examined in this study exhibited either drought avoidance or drought tolerance. The results suggest that *Z. insignis*, identified as the most resilient tree species, predominantly employs a drought avoidance strategy [43,44]. This is evidenced by the rapid reduction in crown width and stomatal conductance, which likely serves as a critical hydraulic safety mechanism to prevent xylem cavitation [45,46]. Such a strategy is characteristic of species adapted to seasonally dry habitats, where high evaporative demand necessitates strict control over the plant water status [47]. Under drought stress, *E. fordii*, *D. odorifera*, *C. burmanni*, and *M. chapensis* exhibited leaf rolling (Supplementary Figure S1), reduction in crown width, stomatal closure, and decreased transpiration rates. These coordinated responses suggest that these species also employ drought avoidance mechanisms [43,44]. In contrast, late-successional species such as *P. bournei* and *P. zhennan* appear to favor a drought tolerance strategy [43,44]. These species maintained relatively higher Gs during the initial stages of drought stress but relied heavily on a robust antioxidant defense to stabilize metabolic functions [48,49]. Specifically, they exhibited significant changes in Pro, MDA, and GSH contents, as well as in the activities of SOD, POD and APX under drought stress. Evolutionarily, this response likely stems from their ancestral niche in moist, competition-heavy valley environments, where maintaining photosynthesis despite declining water availability is crucial for competitive persistence [50,51]. However, the exacerbated accumulation of MDA in these species during late-stage drought suggests that this biochemical response is insufficient to mitigate cumulative oxidative stress, particularly when compared to the effective morphological avoidance exhibited like *Z. insignis* [52]. Therefore, the drought resistance mechanisms may reflect how each species balances water safety and internal defense according to its natural habitat [53].

Drought resistance is not a product of discrete traits but emerges from an integrated physiological network centered on reactive oxygen species (ROS) homeostasis [54,55]. ROS can act as central signaling hubs that integrate multiple pathways to coordinate plant stress responses [55,56]. The observed synchronized reduction in LRWC, Pn, and Gs likely triggers an increase in ROS production by disrupting the chloroplast electron transport chain [32,57]. PCA results suggest CI_1_ as the primary content of this synergistic defense. Specifically, the significant negative correlation between antioxidant enzyme activities, like SOD and POD, and MDA levels confirms that a robust ROS scavenging capacity is essential to mitigate oxidative damage to membrane lipids [58,59]. The synergy was most pronounced in *Z. insignis*, where the peak in SOD activity coincided with a suppressed MDA accumulation, allowing for faster physiological recovery upon rewatering. Moreover, the strong coupling between morphological traits and osmotic solutes (Pro and SP), as revealed by Mantel tests, may reflect a functional synergistic integration aimed at maintaining cellular homeostasis under stress [60]. Under severe drought (D14), sensitive species like *M. chapensis* suffered the steepest Pn declines (90.4%), likely because their antioxidant synergy failed to compensate for the metabolic constraints imposed by leaf curling and reduced crown width [20,49]. APX, in particular, functions synergistically with GSH through the ascorbate–glutathione cycle to maintain cellular redox homeostasis and suppress ROS accumulation [61]. Collectively, these interactions form a complex physiological regulatory network centered on ROS, integrating photosynthesis, antioxidant systems, osmotic regulation, and nutrient metabolism to ensure plant survival under diverse environmental conditions.

Our study of the eight tree species shows that the process of drought-induced damage and the subsequent recovery do not follow the same pattern or speed. The differential sensitivity of species to drought stress leads to varying response times, indicating that species may simultaneously be in different stages of stress response [62,63]. The results demonstrate that the best performing resilience of *Z. insignis* is primarily attributed to its highly responsive avoidance, where the rapid closure of Gs during early drought effectively preserved its internal water balance [37]. The rapid Pn recovery in *Z. insignis* upon rewatering may be attributed to the significantly higher soil relative water content which maintained during the stress period. In contrast, species like *P. bournei* relied on a strong biochemical response, where proline levels increased significantly from 10.6 to 110.2 µg/gFW to help the cells hold onto water [60]. The fact that APX activity and total chlorophyll content remained low during rewatering suggests that the 14-day drought might have caused some degree of lasting oxidative stress in the tissues [56,62]. Drought resistance is also a complex quantitative trait controlled by multiple genes and environment conditions [64,65]. We believe that leaf relative water content, Pn, SOD and MDA may be significant determinants of drought resistance, which is consistent with previous studies [66]. Overall, the results indicate that plants with a quick response to avoid water loss have a clear advantage in recovering their functions after drought.

While this study provides a clear drought resistance ranking and identifies key physiological traits for one-year-old seedlings, extrapolating these results to mature trees in natural forests requires careful consideration. Seedling-level results, inherently influenced by immature root architectures and constrained resource buffering, may fail to fully capture the complex stress experienced by mature trees in natural field conditions [67]. However, seedling-stage screening remains highly relevant for afforestation. The establishment phase is a critical demographic bottleneck for plant populations, and seedling survival is pivotal for successful forest restoration [68,69]. This is especially urgent in the context of extreme weather events, posing significant threats to ecosystems [70]. Therefore, our findings provide a scientific foundation for precision silviculture in Guangdong’s afforestation programs [71]. We recommend the prioritization of *Z. insignis* and *M. macclurei* for reforestation in seasonally arid regions or degraded sites, whereas species with higher drought sensitivity should be reserved for sites with better microclimatic buffering. Future research should integrate multi-scale perspectives to bridge the gap between seedling-level findings and large-scale silvicultural practices [62,72]. Such an approach will yield more intuitive and accurate data, ensuring the scientific effectiveness of practical afforestation applications.

## 4. Materials and Methods

### 4.1. Plant Material and Treatments

One-year-old seedlings of eight native tree species were selected as experimental materials: *Z. insignis*, *P. bournei*, *M. macclurei*, *P. zhennan*, *E. fordii*, *D. odorifera*, *C. burmanni* and *M. chapensis*. The experiment was conducted for a 29-day period in an indoor greenhouse at Tianhe District, Guangzhou, Guangdong, China (113.36° E, 23.16° N) in October 2024. Environmental conditions included a mean air temperature of 28 °C (range: 24–34 °C) and a mean monthly sunshine duration of 177.6 h, as recorded by the Guangdong Provincial Meteorological Service. Before treatments, healthy seedlings of uniform size were selected and watered normally for one week to recover growth. Subsequently, irrigation was withheld to induce natural soil desiccation and establish drought stress gradients (ensuring thorough watering one day prior to drought stress treatment). Following the physiological framework of Flexas (2004) [73], the point of zero net photosynthetic rate (Pn = 0) was designated as the critical threshold for the transition from drought stress to rewatering. Preliminary trials (20 days) revealed that the eight species reached *Pn* = 0 between the 12th and 17th day. Due to interspecific differences, sensitive species approached this threshold earlier, while more resilient trees sustained positive Pn until the 17th day. Ultimately, by integrating the results from our preliminary trials, a 14-day interval was established as the standardized period for the drought stress phases. After 14 days of drought stress, rewatering was initiated.

For each tree species, 120 seedlings were prepared for the experiment. The experimental cohorts were divided as follows: (1) 15 seedlings were reserved for non-destructive morphological measurements; (2) 15 seedlings were used for continuous, non-destructive photosynthetic analysis; and (3) 90 seedlings were allocated for destructive sampling across six critical time points. Leaf samples were collected on D0, D4, D7, D14, R7, R14 (D0 = the day before the drought stress; D4, D7, D14 = the 4th, 7th, and 14th day of drought stress; R7, R14 = the 7th and 14th day of rewatering). At each time point, 15 seedlings were randomly selected. Functional leaves (the third to fifth leaves from the apical portion of the branch) were collected at 9:00 a.m., flash-frozen in liquid nitrogen, and stored at −80 °C. Leaves were excised at the petiole, placed in labeled ziplock bags, flash-frozen in liquid nitrogen, and stored at −80 °C for physiological and biochemical analyses. In the 15 seedlings selected for morphological characterization, plant height, ground diameter and crown breadth were measured using a tape measure, vernier caliper, and ruler.

### 4.2. Soil Relative Water Content and Leaf Relative Water Content

The experimental soil from eight tree species was krasnozem collected from Guangdong Province. Before the commencement of the experiment, the soil field capacity (FC) was determined using the standard cutting ring method [73]. Soil water content was measured (SMC) using a soil hygrometer at a depth of 5 cm near the root zone of three randomly selected seedlings per species. The value was recorded 3 times to determine the average value. Soil relative water content (SRWC) was calculated using the formula SRWC = (SWC/FC) × 100%. During the drought phase, SWC was recorded at four specific sampling time points (D0, D4, D7, and D14). During the rewatering phase, soil moisture was monitored daily to ensure the precision of the moisture gradient recovery, maintaining the soil moisture at approximately 80% of the field capacity.

The fresh weight (FW) of leaves was measured immediately after sampling. The leaves were then submerged in distilled water and kept in darkness for 24 h to achieve turgid weight (TW). Excess surface water was blotted before weighing. The leaves were oven-dried at 105 °C for 0.5 h, followed by 80 °C for 72 h to obtain a constant dry weight (DW). The RWC of the leaves was calculated according to the formula LRWC (%) = (FW − DW/TW − DW) × 100% [74].

### 4.3. Chlorophyll and Photosynthetic Parameters

Leaf samples were cut into 1.0 cm × 0.2 cm strips and homogenized under dim light, and 0.20 g of tissue was immersed in 95% ethanol for 24 h in darkness to extract chlorophyll. Chlorophyll content was determined spectrophotometrically at 470, 649, and 665 nm [75]. The upper, healthy, sunny-side function leaves which are the third to fifth leaves from the apical portion of the branch were measured using a LI-COR 6400 system (Li-COR, Lincoln, NE, USA). The airflow rate was maintained at 500 μmol/s, and the data were read after 2 min of stabilization to record the net photosynthetic rate (Pn), transpiration rate (Tr) and stomatal conductance (Gs) between 8:30 and 11:30 a.m. Water use efficiency (WUE) was calculated using the following formula: WUE (μmol CO_2_/mmol H_2_O) = Pn/Tr. For each tree species, three biological replicates were conducted, with each replicate consisting of 12 individual plants.

### 4.4. Measurements of Osmotic Solutes

Frozen leaf samples were finely ground using a mortar and pestle in an ice bath. Leaf tissue was homogenized in 2 mL of 3% sulfosalicylic acid and centrifuged at 10,000× *g* for 10 min at 4 °C. And proline (Pro) content was quantified using the ninhydrin colorimetric method [76]. Leaf tissue was extracted with 5 mL of 80% ethanol at 80 °C for 30 min, then centrifuged at 4000× *g* for 10 min, and soluble sugar (SS) and starch (Sta) were analyzed via the sulfuric acid–anthrone method, with starch hydrolyzed using α-amylase and amyloglucosidase [77]. Soluble protein (SP) content was determined using the Coomassie Brilliant Blue G-250 staining method after extracting 0.2 g tissue in 2 mL of 50 mM phosphate buffer (pH 7.0) [78]. Each measurement was performed with six biological replicates and three technical replicates to ensure statistical reliability.

### 4.5. Antioxidant Enzyme Activity and Malondialdehyde (MDA) Content

Frozen leaf samples were finely ground using a mortar and pestle in an ice bath. Leaf tissue was homogenized in 3 mL of ice-cold 50 mM phosphate buffer (pH 7.8) containing 1 mM EDTA and 2% (*w*/*v*) polyvinylpyrrolidone. The homogenate was centrifuged at 12,000× *g* for 20 min at 4 °C, and the supernatant was used immediately for enzyme assays. Superoxide dismutase (SOD) activity was measured using the nitroblue tetrazolium reduction method [79]. Peroxidase (POD) activity was assessed spectrophotometrically by monitoring guaiacol oxidation at 470 nm [80]. Leaf tissue was homogenized in 2 mL of 10% trichloroacetic acid, and malondialdehyde (MDA) content was determined via the thiobarbituric acid reaction [80]. Ascorbate peroxidase (APX) activity was evaluated by monitoring ascorbate oxidation at 290 nm [81], and glutathione (GSH) content was analyzed using the DTNB colorimetric method at 412 nm after extracting tissue in 5% metaphosphoric acid [82]. Each measurement was performed with six biological replicates and three technical replicates to ensure statistical reliability.

### 4.6. Data Analysis

All experimental data underwent normality testing (Shapiro–Wilk test) and homogeneity of variance verification (Levene’s test). Data entry, preliminary processing and normalization analysis were performed using Microsoft Excel 2010, and statistical analysis was carried out using SPSS 19.0 (IBM Corp., Armonk, NY, USA). One-way analysis of variance (ANOVA) was applied, and treatment means were compared using Duncan’s multiple range test at *p* < 0.05. Mantel tests and correlation heatmap analyses were carried out using ChiPlot tools “https://www.chiplot.online(accessed on 26 November 2025)”. For principal component analysis (PCA), the number of principal components is typically selected such that the cumulative eigenvalue contribution exceeds 85%. The PCA biplots were generated using Origin Pro 2022 (OriginLab Corp., Northampton, MA, USA). The drought tolerance of eight native tree species was evaluated via a membership function method. When the indicator was positively correlated with drought resistance, the formula was U(X_ij_) = (X_ij_ − X_jmin_)/(X_jmax_ − X_jmin_). When the indicator was negatively correlated with drought resistance, the formula was U(X_ij_) = 1 − (X_ij_ − X_jmin_)/(X_jmax_ − X_jmin_). The comprehensive evaluation value was calculated as D = ∑U(X_ij_) × W_j_). In the formula, X_ij_ is the measured value; X_jmax_ and X_jmin_ represent the maximum and minimum values of each indicator respectively; W_j_ represents the weight of the comprehensive indicator among all the comprehensive indicators; D represents the comprehensive drought resistance evaluation value of different species under drought conditions, obtained through comprehensive index assessment.

## 5. Conclusions

Against the backdrop of ongoing climate change, Guangdong Province is experiencing an intensification in both the frequency and severity of droughts during the dry season (November to March). Consequently, these escalating seasonal water deficits pose an increasingly significant challenge to the survival and establishment rates of seedlings. This study evaluated 21 physiological and biochemical indicators of eight native tree species under drought and rewatering treatments across six stages. The results show that all species exhibited a coordinated response during the initial phase of drought stress: the crown breadth, ground diameter, chlorophyll content, transpiration rate, net photosynthetic rate and stomatal conductance decreased, whereas the soluble protein, proline, malondialdehyde, peroxidase and superoxide dismutase increased. However, these adjustments were insufficient to prevent stress. Drought stress markedly reduced crown breadth, leaf relative water content, chlorophyll content, and ascorbate peroxidase activity, and these parameters did not return to pre-stress levels after rewatering. Interspecific differences in recovery capacity and drought resistance were also pronounced. Multivariate statistics revealed a tightly integrated network among physiological and biochemical characters. A strong correlation exists between photosynthetic parameters and morphological traits, indicating that these indicators act synergistically to determine whole-plant drought resistance. Four principal components were extracted from the 21 physiological and biochemical indicators. The leaf relative water content, net photosynthetic rate, superoxide dismutase and malondialdehyde were identified as the pivotal drought resistance traits, which offers suggestions for future investigations into drought resistance mechanisms. And the comprehensive evaluation score was calculated using membership function analysis. *Z. insignis*, ranked as the most drought-resistant species, employs a drought avoidance strategy characterized by rapid stomatal closure (Gs declining from 1.3 to 0.1 mol/m^2^/s), effective maintenance of soil moisture, and exceptional recovery capacity (Pn recovering to 3.5 μmol CO_2_/m^2^/s by R14). This morphological and physiological coordination provides strong resistance to severe water stress. The resulting composite index yielded the following ranking of drought resistance (highest to lowest): *Z. insignis*, *P. bournei*, *M. macclurei*, *P. zhennan*, *E. fordii*, *D. odorifera*, *C. burmanni* and *M. macclurei*. This offers guidance for precision silviculture. Specifically, *Z. insignis* and *M. macclurei* should be prioritized for drought-prone sites and degraded lands, while species with lower drought resistance should be reserved for locations with reliable moisture or microclimatic protection.

## Figures and Tables

**Table 2:** Principal component analysis of various indexes of eight native tree species.

Measured Index	Principal Component			
	CI_1_	CI_2_	CI_3_	CI_4_
Tota eigenvalue	6.346	5.576	2.623	1.620
Contribution rate (%)	33.402	29.349	13.805	8.528
Cumulative contribution rate (%)	33.402	62.751	76.556	85.084

**Table 3:** Principal component loading matrix of physiological and biochemical characters for eight native tree species.

	CI_1_	CI_2_	CI_3_	CI_4_
SRWC	−0.691	−0.427	−0.157	0.052
PH	−0.674	0.501	−0.019	0.177
GD	−0.65	−0.648	0.088	−0.051
CB	0.651	−0.704	0.127	0.167
LRWC	−0.76	0.195	−0.26	0.319
Chl	−0.206	0.457	0.783	0.026
Pn	0.785	−0.358	−0.125	0.005
Tr	0.136	0.837	−0.168	0.414
WUE	−0.145	−0.78	0.151	−0.528
Gs	−0.153	0.849	−0.257	0.062
Pro	0.586	0.515	0.564	−0.121
SP	−0.436	0.298	0.595	0.1
SOD	0.951	0.087	−0.001	0.127
MDA	−0.67	−0.406	0.225	0.402
POD	−0.226	0.05	−0.885	0.102
APX	0.733	0.478	0.143	0.316
GSH	0.771	−0.25	−0.364	0.166
SS	0.398	−0.761	0.104	0.492
Sta	0.21	0.65	−0.303	−0.646

SRWC: soil relative water content; PH: plant height; GD: ground diameter; CB: crown breadth; LRWC: leaf relative water content; Chl: chlorophyll concentration; Pn: net photosynthetic rate; Tr: transpiration rate; WUE: water use efficiency; Gs: stomatal conductance; Pro: proline; SP: soluble protein; SOD: superoxide dismutase; MDA: malondialdehyde; POD: peroxidase; APX: ascorbate peroxidase activity; GSH: glutathione activity; SS: soluble sugar; Sta: starch.

**Table 4:** Membership function values of eight native tree species.

Species	Membership Function Value					Sequencing
	U_1_	U_2_	U_3_	U_4_	D	
*Z. insignis*	1.830	3.174	0.500	0.332	1.836	1
*M. macclurei*	1.278	1.524	−0.678	−0.454	0.820	2
*P. zhennan*	1.110	−1.659	1.448	−0.057	0.261	3
*P. bournei*	−1.571	3.009	0.312	−0.492	0.233	4
*E. fordii*	−2.044	1.162	1.354	−0.512	−0.310	5
*D. odorifera*	−1.681	1.066	0.051	0.219	−0.373	6
*C. burmanni*	−2.666	1.536	−1.314	0.301	−0.913	7
*M. chapensis*	−3.248	0.301	0.572	0.455	−1.143	8
W_j_	0.43	0.29	0.19	0.09		

W_j_ represents the weight of the comprehensive indicators; D represents the comprehensive drought resistance evaluation value of different species.

## Data Availability

The original contributions presented in this study are included in the article/Supplementary Material. Further inquiries can be directed to the corresponding author.

## Supplementary Materials

The following supporting information can be downloaded at: https://www.mdpi.com/article/10.3390/plants15040528/s1, Figure S1. Leaf curling phenotypes of four tree species under drought stress and rewatering treatments.

## Abbreviations

SMC: Soil moisture content; LRWC: Leaf relative water content; Pn: Net photosynthetic rate; Tr: Transpiration rate; Gs: Stomatal conductance; WUE: Water use efficiency; Pro: Proline; SP: Soluble protein; SS: Soluble sugar; Sta: Starch; GB: Glycine betaine; SOD: Superoxide dismutase; POD: Peroxidase; APX: Ascorbate peroxidase activity; GSH: Glutathione activity; MDA: Malondialdehyde; PCA: Principal component analysis; ROS: Reactive oxygen species.
