# Supplementary material for: Physiological and Biochemical Characters of Eight Native Tree Seedings in Guangdong Province During Drought Stress and Rewatering Treatment
Source: Plants (Basel). 2026 Feb 8;15(4):528. doi: 10.3390/plants15040528 (PMC12944557; doi:10.3390/plants15040528)
Supplement: Supplementary file 1 [file plants-15-00528-s001.zip › plants-4091743-supplementary.pdf]

plants

Supplementary Materials

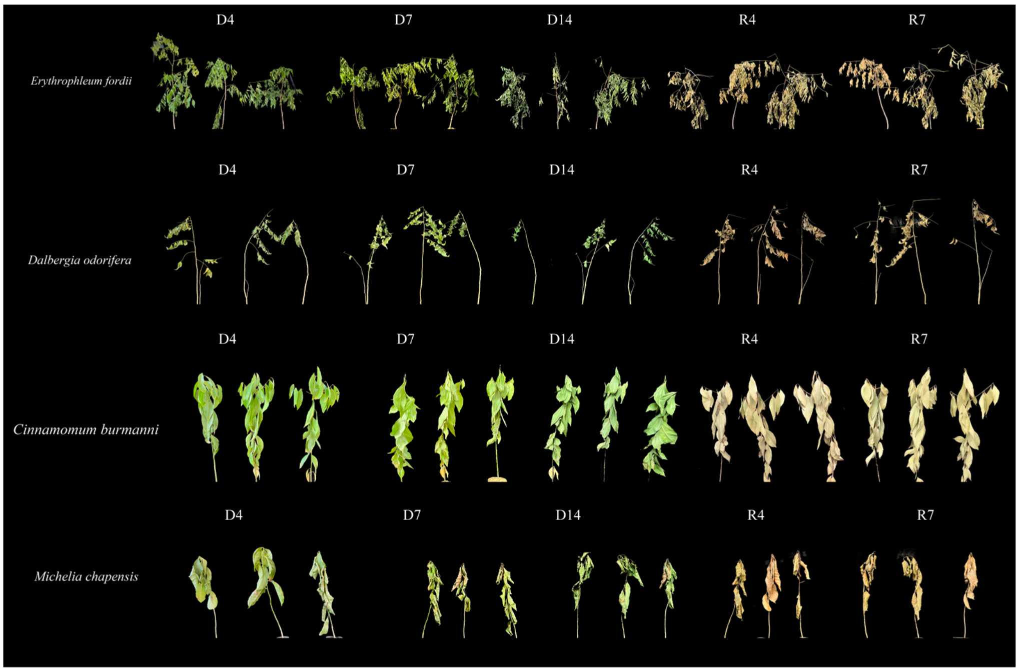

Figure S1. Leaf curling phenotypes of four tree species under drought stress and rewatering treatments.
